# Water Sorption Properties and Hydrothermal Stability of Al-Containing Metal–Organic Frameworks CAU-10 and MIL-96 Studied Using Quasi-Equilibrated Thermodesorption

**DOI:** 10.3390/molecules29235625

**Published:** 2024-11-28

**Authors:** Waclaw Makowski, Patrycja Gryta, Gabriela Jajko-Liberka, Monika Cieślik-Górna, Aleksandra Korzeniowska

**Affiliations:** 1 Faculty of Chemistry, Jagiellonian University in Kraków, Gronostajowa 2, 30-387 Kraków, Poland; patrycja.gryta@doctoral.uj.edu.pl (P.G.); gabriela.jajko@uj.edu.pl (G.J.-L.); monika.cieslik@uj.edu.pl (M.C.-G.); aleksandra.korzeniowska@uj.edu.pl (A.K.); 2 Doctoral School of Exact and Natural Sciences, Jagiellonian University in Kraków, Łojasiewicza 11, 30-348 Kraków, Poland

**Keywords:** adsorption, thermodesorption, water, metal–organic framework, hydrothermal stability, quasi-equilibrated temperature-programmed desorption and adsorption

## Abstract

A novel experimental technique, quasi-equilibrated temperature-programmed desorption and adsorption (QE-TPDA), was used to study the water sorption properties and hydrothermal stability of aluminum trimesate MIL-96 and aluminum isophthalate CAU-10, which have been selected due to their remarkable sorption properties. The QE-TPDA profiles of water observed for MIL-96 and CAU-10 confirmed the hydrophilic nature of these materials. Complex QE-TPDA profiles indicate that water sorption in MIL-96 follows a three-step pore filling mechanism. The shape of single desorption peaks in the QE-TPDA profiles for CAU-10 confirms that water sorption involves a reversible phase transition. Based on the QE-TPDA profiles, the water adsorption heat was determined: 45–46 kJ/mol for CAU-10 and 43–56 kJ/mol for MIL-96, in the latter case depending on the adsorption extent. Hydrothermal stability tests revealed that MIL-96 retained its stable porosity-related sorption capacity for water after hydrothermal treatment up to 290 °C. Gradual changes in the QE-TPDA profiles due to the hydrothermal treatment above 290 °C, with decreasing the high-temperature desorption peak and increasing the low-temperature one, indicate minor structural changes occurring in this material. Only after 410 °C treatment was fast degradation of MIL-96 observed. CAU-10 exhibited high and unchanged hydrothermal stability up to 400 °C.

## 1. Introduction

Metal–organic frameworks (MOFs) are a new generation of porous materials. They consist of metal cations (or their clusters, chains, or layers) linked together into a three-dimensional framework through organic linkers. The MOF crystal lattices contain open voids that are filled during synthesis with the solvent molecules, and in many cases, they remain stable after solvent removal. Because of the presence of the structural pores, MOFs act as molecular sieves, as they can adsorb relatively small molecules of size and shape matching geometry of the pores. MOF materials show great diversity, with the number of known structures probably exceeding a hundred thousand, which is due to the large number of building units (metal cations and linkers) and various possibilities of their connections. Furthermore, for a given type of framework, there are virtually unlimited possibilities to create new structures by modification of the original ligands or using homologous ligands of different sizes [1].

Due to their structural diversity and versatile surface chemistry, MOFs are considered promising materials for numerous potential technological applications, including catalysis, separation, gas storage, drug delivery, atmospheric water harvesting, adsorption-based chillers and heaters, etc. [2,3]. The composition of the feed and typical process conditions used in most of these applications indicate that the materials used will be exposed to water vapor, sometimes at considerably high temperatures. Hence, characterization of water adsorption properties and hydrothermal stability seems essential in any application-oriented study on MOFs [4,5,6,7]. For applications based on adsorption of water, a detailed understanding of the mechanism of water adsorption in the MOF framework seems necessary.

In an influential paper on water adsorption in MOFs, Kaskel et al. [8] showed that water sorption measurements may provide information not only on surface area, pore volume and pore size, but also about surface chemistry and stability of the material toward moisture. For water, which exhibits strong intermolecular interaction, type III isotherms are expected. However, in the case of porous materials, adsorption is limited to the available pore volume; hence, type V isotherms or combinations of type I and III are often observed. The adsorption isotherms of water on MOFs are also affected by the coordination state of the metal cations, presence of polar oxygen bridges in the metal cation clusters and hydrophobicity of the organic linker [8]. The mechanism of water adsorption in MOFs can involve reversible and continuous pore filling, irreversible and discontinuous pore filling (capillary condensation) and/or structural transformation of the framework [9].

Water adsorption measurements are experimentally demanding—they require advanced equipment, either manometric or gravimetric systems with a vapor option. Static manometric measurements may be time consuming due to diffusion limitations resulting from lateral interactions of H_2_O molecules. Dynamic vapor sorption (DSV) measurements, performed using a thermobalance purged with a carrier gas containing a controlled level of the adsorptive, are not considerably faster. Characterization of the MOFs’ hydrothermal stability is also quite complicated as it involves probing the crystalline structure or porosity (by XRD or N_2_ adsorption) before and after exposure of the material to water vapor at a controlled temperature and partial pressure. Taking into account these difficulties, we consider it important to find alternative experimental methods permitting easier and faster measurements of water adsorption profiles in MOFs and characterization of their hydrothermal stability. One such method is quasi-equilibrated temperature programmed desorption and adsorption (QE-TPDA), developed by W. Makowski [10].

Thermodesorption of volatile compounds under quasi-equilibrium conditions may be studied in a flow system equipped with a chromatographic thermal conductivity detector (TCD) using helium containing a small admixture of the adsorptive as a carrier gas. QE-TPDA measurements are performed by cyclic heating and cooling of a quartz tube containing a small sample of the studied sorbent (typically 2–10 mg), through which the carrier gas flows. The QE-TPDA profiles contain desorption maxima observed during heating of the sample and adsorption minima recorded during cooling. Quasi-equilibrium control of thermodesorption may be confirmed by integration of desorption and adsorption peaks. If the integral profiles are close, their average represents the isobar of adsorption (corresponding to the inlet partial pressure of the adsorptive in the carrier gas).

The QE-TPDA of n-alkanes has been successfully applied to the characterization of zeolites [10,11,12,13,14], mesoporous silicas [15,16] and their carbon replicas [16]. Recently, the QE-TPDA method was used in studies on water sorption in STAM-1 [17], UiO-66 [18,19], JUK-8 [20], Al-fum and MIL-88a [21] as well as in porous CN-bridged coordination polymers, comprising Ni and W or Cr or Fe [22]. The aim of this work was to apply the QE-TPDA methodology for characterization of the sorption of water and hydrothermal stability of two selected Al-containing MOFs. Aluminum isophthalate CAU-10 is regarded as a promising water sorbent due to its considerable capacity and excellent stability [23]. Aluminum trimesate MIL-96 exhibits exceptionally high capacity for simultaneous H_2_O and CO_2_ adsorption, which makes it a prospective sorbent for carbon capture and storage technology [24]. This study was specifically focused on better understanding the differences between water adsorption patterns (i.e., isotherms and thermodesorption profiles) observed for both investigated MOFs.

## 2. Results and Discussion

**Figure 1:**
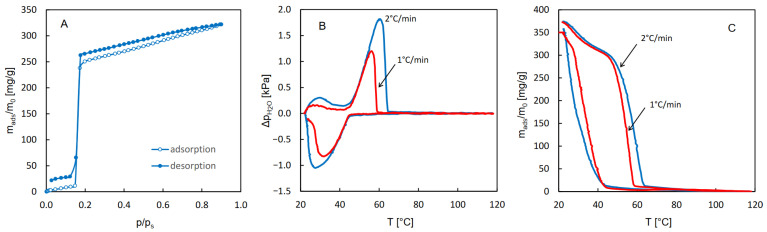
The isotherms of water adsorption and desorption measured at 25 °C (**A**) and QE-TPDA profiles (**B**) of water observed for CAU-10, as well as the integral adsorption and desorption curves (**C**), based on the thermodesorption profiles. Experimental details of QE-TPDA: inlet partial pressure of water p_in_ = 1.9 kPa, sample mass m = 3.3 mg, carrier gas (He) flowrate F = 7.2 cm^3^/min.

The isotherms of water adsorption and desorption as well as the QE-TPDA profiles of water obtained for CAU-10 are shown in Figure 1. Partially overlapping thermodesorption profiles recorded at different heating and cooling rates are characteristic of sorption-induced phase transitions. A similar behavior was previously observed in the case of a CN-bridged Ni-W coordination polymer, exhibiting a single-crystal-to-single-crystal phase transition [25], triggered at 20 °C by water vapor at p/p_s_ = 0.3. The step at p/p_s_ = 0.15, dominating the water sorption isotherms observed for CAU-10, was attributed earlier [24] to a transition between the centrosymmetric phase (dry) and the non-centrosymmetric phase (hydrated), differing in the conformations of the AlO_6_ groups and isophtalate linkers. The desorption patterns presented in Figure 1A,B also indicate high reversibility of adsorption and desorption, with no noticeable hysteresis. This is easy to notice in the case of the isotherms, as they overlap in the step region. For the QE-TPDA profiles, the reversibility of the desorption and adsorption related to the phase transition is indicated by the fact that the overlapping parts of the maxima and minima of the QE-TPDA profiles seem to follow one smooth line.

**Figure 2:**
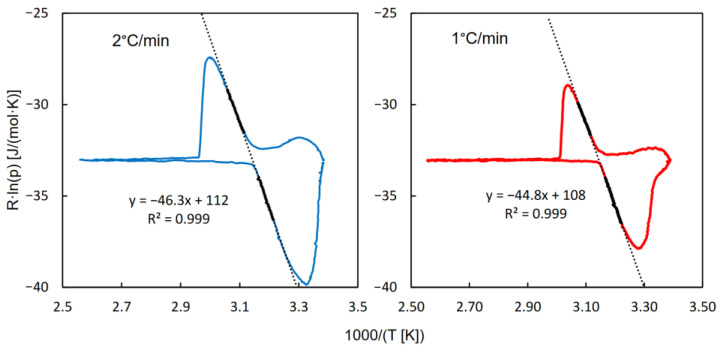
The QE-TPDA profiles from Figure 1B transformed according to the van’t Hoff equation. The formulae in the insets indicate the values of the adsorption enthalpy and entropy and correlation coefficients obtained by linear regression of overlapping fragments of the profiles.

A more convincing graphical representation of this observation is shown in Figure 2, which contains the QE-TPDA profiles transformed according to the van’t Hoff equation. For a reversible phase transition, in which water vapor is the only gaseous reactant, its partial pressure may be treated as an equilibrium constant (K_des_) that obeys the van’t Hoff equation:

(1) $$R\ln\frac{p_{H_{2}O}}{p^{o}}=-\frac{{∆H}_{des}}{T}+{∆S}_{des}$$

It should be noticed that in each plot shown in Figure 2, the linear parts corresponding to the fragments of the QE-TPDA desorption maximum and adsorption minimum may be fitted with a single linear function with remarkable accuracy. Moreover, the absolute values of their slopes (45–46 kJ/mol), only slightly higher than the vaporization heat of water (42–44 kJ/mol), may be regarded as a good measure of the desorption heat. These values are slightly lower than those corresponding to the isosteric adsorption heats, calculated earlier from the adsorption (50–56 kJ/mol) and desorption (52–60 kJ/mol) isotherms [24]. However, it should be noted that the isotherms used in these calculations exhibit considerable hysteresis [26]. Taking into account a complete lack of adsorption–desorption hysteresis in our results, one may expect that the isotherms reported previously [20] have been affected by diffusion limitations.

**Figure 3:**
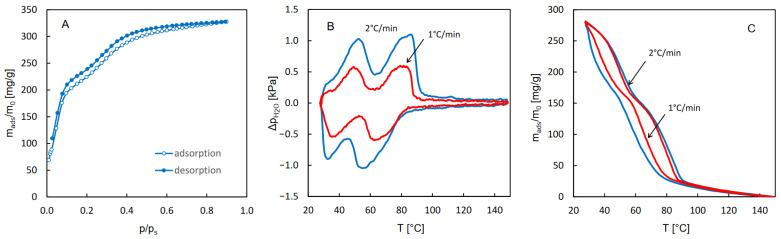
The isotherms of water adsorption and desorption (**A**) and QE-TPDA profiles (**B**) of water observed for MIL-96, as well as the integral adsorption and desorption curves (**C**), based on the QE-TPDA profiles from B. Experimental details of QE-TPDA: inlet partial pressure of water p_in_ = 2.4 kPa, sample mass m = 5.7 mg, carrier gas (He) flowrate F = 7.2 cm^3^/min.

The isotherms of water adsorption and desorption as well as the QE-TPDA profiles of water obtained for MIL-96 are shown in Figure 3. These patterns are completely different from those observed for CAU-10, although they also exhibit high reversibility of adsorption and desorption. While the isotherms practically overlap throughout the relative pressure range, the desorption maxima in the thermodesorption profiles are similar to the mirror reflections to adsorption minima, relative to the horizontal line at p_H2O_ = 0. However, the positions of the maxima and minima are shifted to higher and lower temperatures, respectively. These shifts are intrinsic to the QE-TPDA method and rise with the increase in the heating/cooling rate. The integral desorption and adsorption profiles obtained from the QE-TPDA data recorded at 1 °C/min are close to one another, corroborating reversibility of desorption and adsorption. Regrettably, further decrease in the heating/cooling rate results in a much higher noise-to-signal ratio, which makes the integral profiles considerably distorted.

Both the isotherms and the QE-TPDA profiles shown in Figure 1 reveal a complex three-step pattern of water adsorption and desorption, consistent with the experimental and computational findings reported earlier by Benoit et al. [27]. Initial uptake, limited to very low relative H_2_O pressures (p/p_s_ = 0–0.03), is barely noticeable, and is represented by only four points in the adsorption isotherm. This initial uptake has been attributed to adsorption at specific adsorption sites; that is, terminal H_2_O molecules or OH anions bound to Al cations [27]. The first pore-filling step (for p/p_s_ = 0.03–0.12) corresponds to the formation of water clusters in the most hydrophilic cages, where the specific adsorption sites are located. In the second step (for p/p_s_ = 0.12–0.50), the channels containing bridging OH groups are filled [27]. It should, however, be noted that the isotherm computed as a result of the molecular simulations did not replicate the stepwise behavior of the experimental one [27]. The three-step adsorption–desorption pattern is clearly reproduced in the QE-TPDA profiles. The initial uptake, which corresponds to the high temperature range (100–150 °C), is slightly underestimated, probably due to the final desorption temperature being too low.

**Figure 4:**
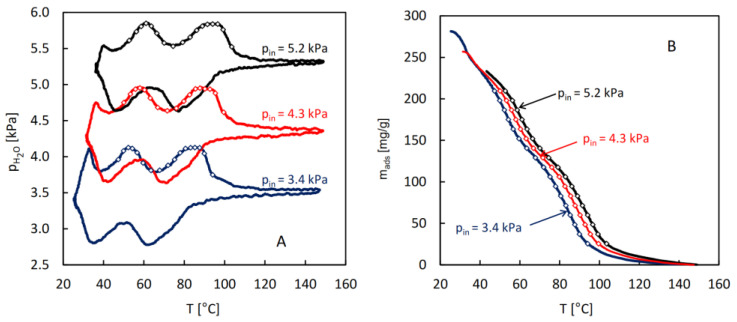
The QE-TPDA profiles (**A**) of water observed in the new experimental system for MIL-96 at a heating/cooling rate of 2 °C/min at different inlet partial pressures, and the corresponding integral desorption curves (**B**). The points indicate data found for the preset adsorbed amount values (from 25.5 to 209.5 mg/g, with 11.5 mg/g interval). Experimental details: sample mass m = 5.5 mg, carrier gas (N_2_) flowrate F = 8.0 cm^3^/min.

**Figure 5:**
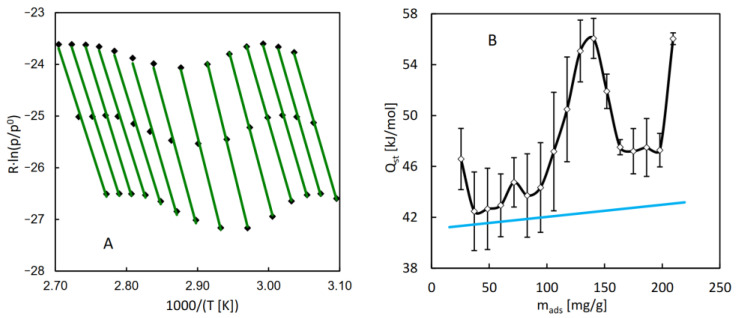
(**A**) The transformed adsorption isosteres of water on MIL-96 calculated from data indicated as points in Figure 4. (**B**) Isosteric adsorption heat of water on MIL-96 obtained from analysis of the adsorption isosteres. The blue line indicates the values of the condensation heat of water, corresponding to the average temperatures of particular isosteres.

For the purpose of the calculation of the isosteric heat of water adsorption in MIL-96, a series of QE-TPDA measurements was performed for different values of the H_2_O partial pressure in the carrier gas (Figure 4A). The integral desorption curves were calculated from the obtained thermodesorption profiles (Figure 4B). Based on these curves and the original QE-TPDA data, 17 isosteres (each consisting of three points) were constructed for the preset loadings in the range of 25–210 mg/g (Figure 5A). The dependence of the isosteric adsorption heat calculated from the slopes of the transformed isosteres on the loading is shown in Figure 5B. The values of adsorption heat are close to the condensation heat (41.4–43 kJ/mol in the relevant temperature range) and are noticeably lower than the low coverage adsorption heat (67 kJ/mol) obtained in molecular simulations [27], corresponding to the Henry adsorption constant. This apparent discrepancy may be justified by the fact that the calculations of the isosteric adsorption heat were limited to the intermediate loading values. Actually, the adsorption heat found for the lowest loading analyzed (corresponding to the points at the end of the initial uptake range) is noticeably higher than the following values.

The fact that the isosteric adsorption heat increases with the increasing loading may seem counterintuitive. However, this behavior may be explained by simultaneous increase in the absolute value of the adsorption entropy (|∆*S_ads_*|). This effect may be expected for sorption of small molecules in small micropores. Initially, the adsorbed molecules exhibit high mobility and little ordering (corresponding to low values of |∆*S_ads_*|) but increasing loading leads to limited mobility and a higher degree of ordering, resulting in higher values of |∆*S_ads_*|. Such behavior was found in the case of n-alkane sorption in the frameworks of MFI and MEL-type zeolites [10,14].

**Figure 6:**
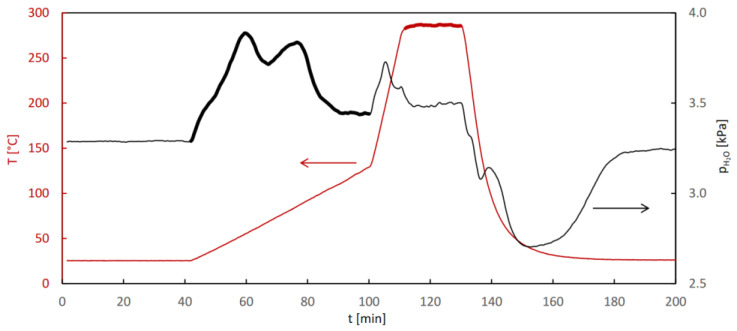
Illustration of the principle of the hydrothermal stability test: the evolution of the sample temperature and the detector signal during a single desorption–adsorption cycle. This example corresponds to the first cycle recorded for MIL-96 (see Figure 7). The diagnostic segment and the hydrothermal treatment segment are indicated by thick black and dark red lines, respectively.

The QE-TPDA measurements were specially adopted for probing the hydrothermal stability of the studied MOFs. A programmable microprocessor system was used for programming complex multi-cycle profiles of temperature changes. Each cycle comprised a diagnostic segment, in which a temperature-programmed desorption signal was recorded at a low heating rate (2 °C/min) and a hydrothermal treatment segment, in which the sample (being purged with the carrier gas containing water vapor) was heated to a high temperature (Figure 6). This temperature was gradually increased (by 20 or 25 °C) every 3 cycles, and the whole stability test consisted of 15–21 cycles.

The results of the hydrothermal stability test performed for MIL-96 reveal unexpected evolution of the thermodesorption profiles after the treatment above 290 °C. A gradual increase in the low temperature desorption peak and decrease in the high temperature one upon increasing the temperature of the hydrothermal treatment were observed. These changes resulted in a small increase in the total sorption capacity after the treatment at 330 °C. The onset of decreasing sorption capacity was noticed at 390 °C, but after the treatment at 410 °C, the desorption intensity dropped quickly, indicating considerable damage of the porous structure of MIL-96.

In order to verify whether these changes in the thermodesorption profiles could be attributed to structural changes of MIL-96, a series of powder XRD measurements was performed for three samples after stability tests, in which the final temperature of the hydrothermal treatment equal to 280, 350 and 290 °C was reached. The observed diffractograms are collated in Figure 8. They all contain the same reflexes but show considerably lower intensity than that obtained for the as-synthesized material. These results indicate that no structural transformation of the MIL-96 framework occurred, but the size of its crystallites decreased upon hydrothermal treatment. The PXRD patterns and the results of the hydrothermal stability tests of MIL-96 seem to contradict the interpretation of the three-step adsorption patterns based on the outcome of molecular simulations [27] discussed earlier. Evidently, the interactions of water molecules located in different sites within the framework depend on the morphology of the MIL-96 crystallites.

The results of the stability test of CAU-10 (Figure 9) show exceptional resistance of this material against hydrothermal damage. The thermodesorption profiles remain unchanged even after hydrothermal treatment at 400 °C. Only after reaching 425 °C may a small decrease in desorption intensity be noticed, followed by almost a complete loss of adsorption capacity after treatment at 450 °C.

## 3. Materials and Methods

MIL-96 was synthesized according to the original method [28] from an aluminum nitrate nonahydrate (>98%, Merck, Darmstadt, Germany) and trimesic (1,3,5-benzenetricarboxylic) acid (H_3_btc, 95%, Merck, Darmstadt, Germany). H_3_btc (2.1104 g) was placed into a 100 mL Teflon-lined steel autoclave, followed by the addition of 40 mL of deionized water under continuous stirring. Separately, Al(NO_3_)_3_·9H_2_O (18.7658 g) was dissolved in 30 mL of deionized water. The resulting solution was then combined with the H_3_btc solution and stirred vigorously for 10 min. The autoclave was subsequently sealed and heated to 210 °C for 24 h under autogenous pressure. After the reaction, the resulting solid product was isolated by filtration, thoroughly washed with deionized water, and left to dry in air at room temperature. The synthesis yield was 43%. CAU-10 was purchased from Strem Chemicals, Newburyport, USA. The PXRD patterns recorded for CAU-10 before and after the QE-TPDA measurements confirm structure and phase purity of this material, as well as its stability during in the thermodesorption experiments (Figure 10).

**Figure 7:**
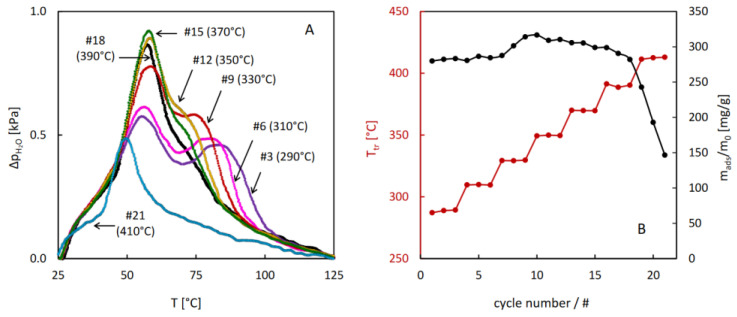
Results of the hydrothermal stability test for MIL-96: selected QE-TPD profiles corresponding to the last cycle in the 3-cycle sequences (**A**) and evolution of the hydrothermal treatment temperature and sorption capacity with the cycle number (**B**). Experimental details: heating rate β = 2 °C/min, inlet partial pressure of water p_in_ = 2.8 kPa, sample mass m = 3.0 mg, carrier gas (N_2_) flowrate F = 8.0 cm^3^/min.

**Figure 8:**
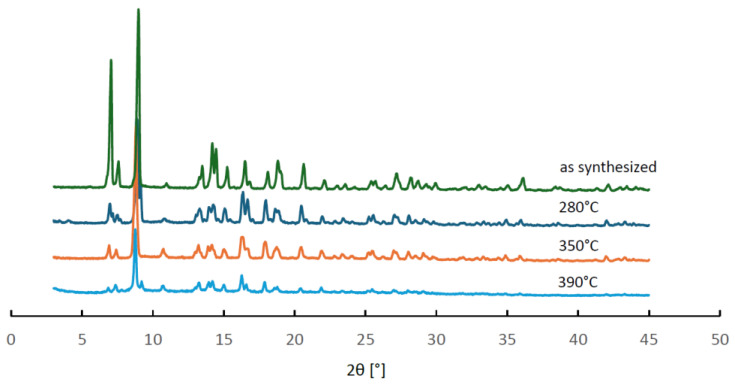
Powder XRD patterns of MIL-96 samples after the QE-TPDA hydrothermal stability tests interrupted at different temperatures compared with the pattern recorded for the as-synthesized sample.

**Figure 9:**
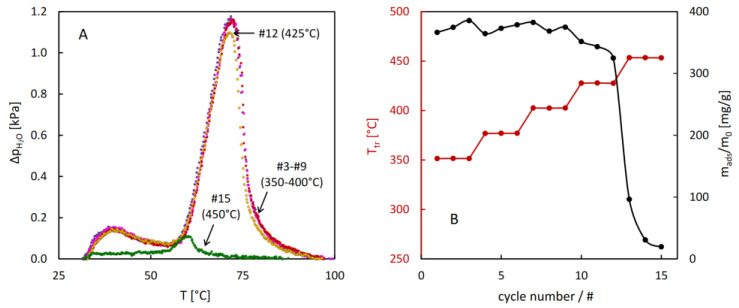
Results of the hydrothermal stability test for CAU-10: selected QE-TPD profiles corresponding to the last cycle in the 3-cycle sequence (**A**) and evolution of the hydrothermal treatment temperature and sorption capacity with the cycle number (**B**). Experimental details: heating rate β = 1 °C/min, inlet partial pressure of water p_in_ = 2.8 kPa, sample mass m = 3.0 mg, carrier gas flowrate F = 8.0 cm^3^/min.

**Figure 10:**
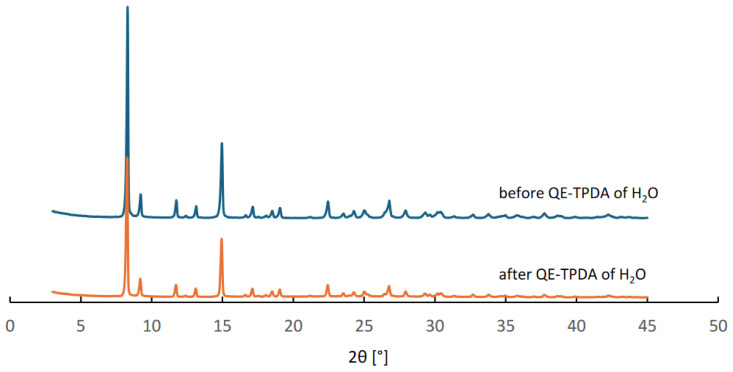
Powder XRD patterns of CAU-10 samples before and after the QE-TPDA of H_2_O measurements.

The standard QE-TPDA system, described earlier [17,18,19], was used in measurements reported in Figure 1 and Figure 3. It contained a dual channel thermal conductivity detector (MicroVolume TCD3, VICI Valco, Schenkon, Switzerland). Helium was used as a carrier gas. The sample was placed in a quartz tube, located in a small electric furnace, controlled by an advanced programmable temperature controller (Shinko PC-935, purchased from ACSE, Kraków, Poland). Additionally, the new low-cost QE-TPDA system, described in detail elsewhere [21], was used in the measurements reported in Figure 4, Figure 6, Figure 7 and Figure 9. It contained a home-made miniature detector, based on the Sensirion STC31 thermal conductivity sensor (STC31 SparkFun, purchased from DigiKey.com). Nitrogen was used as the carrier gas. Compared to the standard one, the new system enabled more precise temperature control of the saturator and the sample chamber, as well as easier programming of complex sample temperature profiles with the use of Arduino microprocessor board.

Autosorb iQ MP (Quantachrome/Anton Paar, Graz, Austria) was used for measurements of the adsorption–desorption isotherms of water vapor. Before the measurements, the samples were evacuated for 6 h at 220 °C. The crystallinity of the studied MOFs was characterized with powder XRD using the Rigaku (Tokyo, Japan) MiniFlex 600 diffractometer at room temperature (295 K) with Cu-Kα radiation (λ = 1.5418 Å) in a 2θ range from 3° to 45° with a 0.02° step and 3°/min scan speed.

## 4. Conclusions

Application of quasi-equilibrated temperature-programmed desorption and adsorption (QE-TPDA) methodology in a study on water sorption in two selected aluminum-containing MOFs (CAU-10 and MIL-96) revealed new and interesting properties of these materials. High reversibility of the centrosymmetric to non-centrosymmetric phase transition responsible for stepwise change in the adsorption isotherm, characterized by the adsorption heat of 45–46 kJ/mol, was observed for CAU-10. The reversible three-step pore filling mechanism of water sorption in MIL-96 was confirmed.

The QE-TPDA was also found as a good method for characterizing hydrothermal stability of MOFs. MIL-96 retained its stable porosity-related sorption capacity for water after hydrothermal treatment up to 290 °C. Gradual changes in the QE-TPDA profiles due to hydrothermal treatment above 290 °C, with decreasing the high-temperature desorption peak and increasing the low-temperature one, indicate minor structural changes occurring in this material. Only after treatment at 410 °C was fast degradation of MIL-96 observed. CAU-10 exhibited high and unchanged hydrothermal stability up to 400 °C.

## Data Availability

The data presented in this study are openly available in RODBUK Cracow Open Research Data Repository at https://uj.rodbuk.pl/dataset.xhtml?persistentId=doi:10.57903/UJ/CRAGGM, accessed on 22 October 2024).
